# Population impact of South Africa's human papillomavirus (HPV) vaccination programme on HPV prevalence in adolescent girls with and without HIV: a repeat cross-sectional study

**DOI:** 10.1016/S2214-109X(25)00525-X

**Published:** 2026-03-16

**Authors:** Dorothy A Machalek, Dorothy C Nyemba, Danielle Travill, Kathy Petoumenos, Zizipho Z A Mbulawa, Ishana Naidoo, Feni M M Motshwane, Lesley Bamford, Helen Rees, John M Kaldor, Sinead Delany-Moretlwe, HOPE study group, Admire Chikandiwa, Mojalefa Makae, Thandiwe Mzimela, Thembisile Mogodiri, Nontokozo Ndlovu, Edwin Mkwanazi, Richard Munthali, Julia ML Brotherton, Andrew J Vallely, Rebecca Guy, Suzanne M Garland, Ian H Frazer, Prisha Balgovind, Gerald L Murray

**Affiliations:** a Kirby Institute, University of New South Wales, Sydney, NSW, Australia; b Centre for Women's Infectious Diseases, The Royal Women's Hospital, Melbourne, VIC, Australia; c Wits RHI, University of the Witwatersrand, Johannesburg, South Africa; d Walter Sisulu University, National Health Laboratory Service, Mthatha, South Africa; e National Department of Health, Pretoria, South Africa; f Department of Paediatrics and Child Health, University of Pretoria, Pretoria, South Africa

## Abstract

**Background:**

A school-based human papillomavirus (HPV) vaccination programme providing protection against types 16 and 18 of HPV was introduced in South Africa in 2014 for Grade 4 girls (aged ≥9 years), achieving 87% coverage among learners with at least one of the two recommended doses. We evaluated the programme's impact on HPV prevalence among adolescent girls in a setting of high HIV prevalence.

**Methods:**

In this repeat cross-sectional study, girls aged 17–18 years were invited from 15 primary health-care clinics in four provinces (Free State, Gauteng, Mpumalanga, and North West) of South Africa to provide a self-collected vaginal sample for HPV testing (AnyPlex II HPV28, Seegene, Seoul, South Korea). A survey done from June 19 to Dec 11, 2019, estimated baseline (pre-vaccine) HPV prevalence. A repeat survey done from Feb 16 to Dec 6, 2023, estimated HPV prevalence in a group eligible for the vaccination programme (post-vaccine). Vaccination status was assessed through district registers and self-reported data. The primary outcome was the impact of the HPV vaccination programme, measured as the relative reduction in HPV prevalence between the two birth cohorts using generalised linear regression (estimating adjusted prevalence ratios), overall and by HIV status.

**Findings:**

Of 2470 participants enrolled, 819 girls were recruited for the pre-vaccine survey (248 living with HIV) and 1538 for the post-vaccine survey (295 living with HIV). Prevalence of HPV vaccine types HPV-16 and HPV-18 declined by 83%, from 21·6% (177 of 819 participants) in the pre-vaccine group to 3·2% (49 of 1538 participants) in the post-vaccine group (adjusted prevalence ratio 0·17, 95% CI 0·12–0·24; p<0·0001). A similar reduction was observed among those living with HIV, with prevalence decreasing from 29·4% (73 of 248 participants) in the pre-vaccine group to 4·4% (13 of 295 participants) in the post-vaccine group (adjusted prevalence ratio 0·18, 95% CI 0·10–0·32; p<0·0001). No significant reductions were noted for other HPV types, except HPV-31 and HPV-45, which is consistent with cross-protection.

**Interpretation:**

In this large-scale evaluation of South Africa's two-dose HPV vaccination programme, we observed impacts similar to those seen with three-dose programmes in high-income settings, including equivalent impacts among adolescent girls living with HIV. These findings underscore the substantial population-level benefits of high-coverage routine HPV vaccination in a high-HIV-burden setting.

**Funding:**

Australian National Health and Medical Research Council and the Gates Foundation.

## Introduction

Cervical cancer is a major public health issue worldwide and the third most common cancer among women of childbearing age, with an estimated 662 301 new cases and 348 874 deaths in 2022.^1^ The burden is disproportionately high in low-income and middle-income countries (LMICs), where over 90% of cases and deaths occur. Poor access to health-care services, including effective screening and treatment, has contributed to this higher burden. The situation is further worsened in countries with high HIV prevalence, as women living with HIV face, on average, a six-fold increased risk of developing cervical cancer compared with women without HIV.^2^

Prophylactic human papillomavirus (HPV) vaccines provide high-level protection against HPV types responsible for 70–90% of cervical cancer cases globally.^3^ These vaccines have been introduced into national immunisation programmes in nearly all high-income countries (98%) and in 67% of LMICs^4^ primarily targeting preadolescent or adolescent girls. Initially approved as a three-dose regimen and adopted mainly by high-income countries, the schedule was revised in 2014 when WHO endorsed a two-dose schedule, supported by immunogenicity bridging data, simplifying delivery and expanding access in LMICs. In 2022, WHO further recommended a single-dose schedule for the general population.^5^, ^6^ However, for individuals living with HIV, three doses remain WHO's recommended approach.^6^

**Research in context**

**Evidence before this study**

Prophylactic human papillomavirus (HPV) vaccines targeting adolescents have shown high population impacts and effectiveness in preventing HPV infection and associated diseases, including cervical cancer. Most evidence comes from high-income countries using three-dose schedules. Evidence is scarce for two-dose programmes delivered in low-income and middle-income countries (LMICs). Data on programme impact among adolescent girls and young women living with HIV are sparse. A pragmatic approach for monitoring impact has been the use of repeated cross-sectional surveys to measure changes in HPV prevalence. We searched PubMed from database inception to June 23, 2025, using the terms “HPV”, “vaccine”, “impact”, and “effectiveness”, with no language to identify studies assessing the impact of HPV vaccine programmes in LMICs. Three studies from Malaysia, Bhutan, and Rwanda evaluated three-dose school-based vaccination programmes targeting girls aged 12–13 years, reporting over 85% reductions in vaccine-targeted HPV types. Two additional studies in Bhutan and Rwanda assessed catch-up vaccination in individuals aged 27–29 years, showing reductions of 93% and 52%, respectively, probably influenced by differences in coverage and age of first sexual activity. The Rwanda study found no impact among women living with HIV, probably because many were sexually active before vaccination. A small case-control study in Thailand reported 100% effectiveness of the two-dose vaccine targeting HPV types.

**Added value of this study**

This repeat cross-sectional study provides the first direct population-level evidence of the impact of the two-dose HPV vaccination programme in South Africa, which has a large population of young women at risk of HIV and cervical cancer. This study quantified vaccine impact by comparing HPV prevalence in eligible and ineligible cohorts, assessed differences by HIV status, and measured indirect effects in unvaccinated girls, offering unique real-world insights in a setting previously under-represented in HPV vaccine post-licensure surveillance research.

**Implications of all the available evidence**

Findings show that HPV vaccination in early adolescent girls (aged 9–10 years) provides strong protection even in high HIV-burden settings, supporting continued investment in routine national programmes across sub-Saharan Africa, where many girls might acquire HIV after vaccination. The results are particularly relevant for countries that have introduced or are planning to adopt the WHO-recommended single-dose schedule in 2022. They highlight the importance of high vaccine coverage, early initiation of antiretroviral therapy for girls with HIV, and sustained surveillance to monitor HPV vaccine impact in diverse real-world settings.

Repeat prevalence surveys have shown that HPV vaccination programmes substantially reduce population-level HPV infection and precancerous lesions.^7^ A 2024 study also found high effectiveness against cervical cancer.^8^ However, most of the evidence comes from three-dose programmes implemented in high-income countries. Only a few LMICs, such as Malaysia, Bhutan, and Rwanda, have reported population-level benefits from adolescent-targeted HPV vaccination programmes.^9^, ^10^ In Rwanda, additional surveillance found no reduction in HPV prevalence following a catch-up programme among adolescent girls and young women (aged 17–29 years) living with HIV.^11^ To date, there is no evidence on the programmatic impact of HPV vaccination delivered in preadolescence in high HIV-burden settings, where some girls might acquire HPV and HIV after vaccination.

South Africa, the sixth most populous country in Africa, has the third highest prevalence of HIV among women of childbearing age (ie, 15–49 years), and 10 532 new cervical cancer cases and 5976 deaths were estimated (age-standardised rates of 33 and 19 per 100 000 women, respectively) in 2022.^1^, ^12^ In 2014, the South African National Department of Health launched a national HPV vaccination programme aimed at Grade 4 girls in public schools (aged **≥**9 years) with two doses of the bivalent HPV vaccine.^13^ The programme achieved 87% coverage among learners with at least one dose in the first year, maintaining above 80% coverage in the programme's initial years.^4^ We evaluated the impact of the programme on community-level HPV prevalence, examined the potential influence of HIV co-infection, and quantified indirect effects among unvaccinated girls and cross-protection against non-vaccine HPV types.

## Methods

### Study design and participants

As previously described,^14^, ^15^, ^16^ the HOPE study was a repeat cross-sectional study comparing HPV prevalence in two independent birth cohorts of adolescent girls aged 17–18 years, recruited from 15 publicly funded primary health-care clinics in four South African provinces (Free State, Gauteng, Mpumalanga, and North West). These clinics are typical of those accessed by approximately 90% of the South African population who rely on free public health care.^17^ The pre-vaccine group was surveyed from June 19 to Dec 11, 2019, and included participants who would have been in Grade 5 when the national HPV vaccination programme began in 2014, and therefore probably missed vaccination. The post-vaccine group was surveyed from Feb 16 to Dec 6, 2023, and included participants who would have entered Grade 4 in 2014–15 (ie, were aged 9–10 years) and were thus eligible for vaccination when the programme began.

Eligibility criteria included female sex, age 17–18 years, attending the participating clinics for routine care (eg, contraception, HIV services, and minor illnesses), and willingness and ability to provide written informed consent. All attendees who met the inclusion criteria were consecutively invited to participate. The project was approved by the University of the Witwatersrand Human Research Ethics Committee (HREC; #181005) and the University of New South Wales HREC (#181–005). All participants provided written informed consent. For participants aged 17 years, parental consent was waived with the review and approval of the HREC due to the study's low risk but sensitive nature.

### Procedures

Recruitment procedures were standardised across sites and surveys. Research staff were trained to obtain written informed consent from all participants. Participants completed a computer-assisted self-interview, including questions about their sociodemographic characteristics (eg, household income), HPV risk factors, and vaccination history. Participants who were not known to be living with HIV and had not had an HIV test in the previous 3 months were offered HIV counselling and testing. Those who tested positive received post-test counselling and were actively referred to care according to national guidelines.^18^ For participants living with HIV, the date of HIV diagnosis, the most recent treatment regimen, viral load, and CD4 cell count (when available) were abstracted from medical records with participant's consent. Following instructions from research staff, participants self-collected a vaginal sample for HPV testing at the clinic using a dry flocked swab (Copan Diagnostics, Brescia, Italy).

Samples were stored at 4°C or less and transported to a central laboratory for processing. Swabs were mixed in 1 mL Digene transport medium (Qiagen, Hilden, Germany) to suspend cellular material. Nucleic acids were extracted from 400 μL of the suspension using the MagNA Pure Compact system and Nucleic Acid Isolation Kit (Roche Molecular Systems, Branchburg, NJ, USA). Extracted specimens were tested and genotyped for HPV DNA using the Seegene Anyplex II HPV28 assay (Seegene, Seoul, South Korea), a multiplex semiquantitative PCR melting-curve test detecting 28 HPV types.^15^, ^16^

### Statistical analysis

The prevalence of HPV types 16 or 18 in South African girls aged 17–18 years was previously estimated at approximately 19%^19^ among those not living with HIV and 30% among those living with HIV.^20^ Assuming these baseline estimates, the study was designed with at least 80% power to detect a post-vaccination prevalence of 11·4% among girls not living with HIV and 18·0% among those living with HIV—a relative reduction of 40%—as significant at the 0·05 level.^14^ This power corresponds to a minimum required sample of approximately 348 participants per survey for girls not living with HIV and 198 participants per survey for girls living with HIV. Meta-analyses of vaccine impact studies from countries that implemented three-dose programmes reported relative reductions in HPV-16 and HPV-18 prevalence among vaccine-eligible girls aged 15–19 years of 72% (adjusted prevalence ratio 0·28, 95% CI 0·19–0·41) when coverage was high (≥50%) and 50% (adjusted prevalence ratio 0·50, 95% CI 0·34–0·74) when coverage was lower (<50%).^7^, ^14^

Records of doses administered in the national programme were sought from National Department of Health district HPV vaccination registers. Approximate string matching was used to determine a match probability (0–100%) between the identifying information (first name, last name, and date of birth) provided by study participants and the records in the school registers. A match probability of 90% or higher was considered valid. The same approach was used to identify girls who might have received a single HPV dose through a one-time catch-up campaign conducted in one of the study provinces in 2021.^14^, ^16^ This campaign was part of a separate component of the project evaluating the impact of a single-dose schedule.^16^ To evaluate the full effect of the HPV vaccination programme, the pre-vaccine survey included only unvaccinated participants; those whose identifying information matched records on the school or single-dose HPV registers were excluded from this analysis. In the post-vaccine group, only participants matched to the single-dose register were excluded; those matched to district vaccination registers, as well as those not matched to either register, were retained for analysis. This approach focused the analysis on the impact of the routine school-based vaccination programme delivered in Grade 4.

Girls were classified as vaccinated if they matched a record in the National Department of Health register indicating receipt of at least one dose of vaccine. To mitigate potential missing data due to incomplete registers, we also assessed the impact based on self-reported vaccination status. Girls without a registered record who reported being vaccinated were classified as vaccinated based solely on self-report. Those with no matching district record who reported being unvaccinated or were unsure were classified as unvaccinated.

We estimated crude HPV prevalence with 95% CIs for: (1) HPV-16 and HPV-18 (the prevention targets of the bivalent vaccine) as a pair and individually; (2) HPV-31 and HPV-45, which are related to HPV-16 and HPV-18 and have been observed at reduced prevalence in cohorts who received the bivalent vaccine, and more recently incorporated in the nonavalent vaccine;^21^ (3) remaining high-risk types HPV-33, HPV-52, and HPV-58 targeted by the nonavalent vaccine; (4) other high-risk types not targeted by the vaccine (ie, HPV-35, HPV-39, HPV-5, HPV-56, HPV-59, and HPV-68); (5) any high-risk HPV type (ie, HPV-16, HPV-18, HPV-31, HPV-33, HPV-35, HPV-39, HPV-45, HPV-51, HPV-52, HPV-56, HPV-58, HPV-59, HPV-68); and (6) low-risk types HPV-6 and HPV-11. The primary outcome was the impact of South Africa's HPV vaccination programme, measured as the relative reduction in HPV prevalence between pre-vaccine and post-vaccine groups, following the definition proposed by Germaine Hanquet and colleagues.^22^ We estimated the relative reduction in HPV prevalence overall and stratified by HIV status. We assessed indirect effects by comparing HPV prevalence in the pre-vaccine group with HPV prevalence in unvaccinated girls in the post-vaccine group. For this analysis, unvaccinated girls were defined as those with no register-confirmed doses and those who self-reported being unvaccinated. In a sensitivity analysis, all girls without register-confirmed doses were classified as unvaccinated, regardless of their self-reported status. We assessed cross-protection by comparing the prevalence of HPV-31 and HPV-45 between pre-vaccine and post-vaccine groups.

We used χ^2^squared tests to compare categorical sociodemographic and behavioural characteristics between study groups. These characteristics were identified a priori as relevant to HPV risk, based on published literature.^14^, ^16^ Subgroup analyses were stratified by HIV status and vaccination status. Binomial log-linear regression was then applied to estimate prevalence ratios with 95% CIs. Adjusted models included variables that differed between groups at a p value less than 0·10. Each HPV prevalence category, as defined above, was modelled separately using this approach. Multicollinearity among covariates was assessed using variance inflation factors derived from a linear regression model including the same covariates. Due to expected collinearity between age at HIV diagnosis and time on antiretroviral therapy (ART)—both derived from ART initiation date—these variables were modelled separately to avoid multicollinearity. Missing values were treated as a separate category in the primary analysis. To assess the robustness of our findings, we also conducted a complete-case analysis excluding participants with missing data. The main analysis included all participants, including those who reported never having had vaginal sex. In sensitivity analyses, we repeated the analysis and restricted to those who reported ever having vaginal sex. Separately, we also estimated differences in the prevalence of individual HPV types between the pre-vaccine and post-vaccine groups. We performed all analyses with Stata, version 18.0.

### Role of the funding source

The funders had no role in the data collection, data analysis, data interpretation, or writing of this report.

## Results

A total of 2470 participants were enrolled across four provinces: 900 in the 2019 pre-vaccine group and 1570 in the 2023 post-vaccine group. In the pre-vaccine group, 75 participants vaccinated during the 2019 single-dose campaign and seven with school-based vaccination records were excluded. Across both cohorts, 31 participants had invalid HPV self-testing samples (four in the pre-vaccine group and 27 in the post-vaccine group). 819 participants in the pre-vaccine group and 1538 in the post-vaccine group were included in the final analysis.

Participants in the post-vaccine group were younger, more likely to be from the Free State, and less likely to smoke, report sexual activity, or condom use at last sexual encounter (table 1). Fewer participants were living with HIV in the post-vaccine group than the pre-vaccine group.

**Table 1:** Cohort characteristics by survey period

		**Pre-vaccine group (n=819)**	**Post-vaccine group (n=1538)**	**p value**
Age		..	..	0·0006
	17 years	396/819 (48·4%)	857/1538 (55·7%)	..
	18 years	423/819 (51·6%)	681/1538 (44·3%)	..
HIV status		..	..	<0·0001
	HIV negative	540/819 (65·9%)	1218/1538 (79·2%)	..
	HIV positive	248/819 (30·3%)	295/1538 (19·2%)	..
	Unknown	31/819 (3·8%)	25/1538 (1·6%)	..
Province of residence		..	..	<0·0001
	Free State	506/819 (61·8%)	1080/1538 (70·2%)	..
	Gauteng	114/819 (13·9%)	154/1538 (10·0%)	..
	Mpumalanga	89/819 (10·9%)	149/1538 (9·7%)	..
	North West	110/819 (13·4%)	155/1538 (10·1%)	..
In school		684/816 (83·8%)	1398/1538 (90·9%)	<0·0001
Head of household has income		..	..	<0·0001
	No	310/798 (38·8%)	286/1538 (18·6%)	..
	Yes	336/798 (42·1%)	1010/1538 (65·7%)	..
	Unknown	152/798 (19·0%)	242/1538 (15·7%)	..
In a relationship		372/819 (45·4%)	726/1538 (47·2%)	0·41
Current smoker		86/797 (10·8%)	75/1526 (4·9%)	<0·0001
Frequency of alcohol use		..	..	<0·0001
	Never	455/818 (55·6%)	694/1428 (48·6%)	..
	Once per month or less	275/818 (33·6%)	613/1428 (42·9%)	..
	Two times per month or more	88/818 (10·8%)	121/1428 (8·5%)	..
Uses contraception		640/819 (78·1%)	919/1538 (59·8%)	<0·0001
Ever had sex		607/816 (74·4%)	904/1468 (61·6%)	<0·0001
Age at first vaginal sex				
	Reported no vaginal sex	220/764 (28·8%)	724/1471 (49·2%)	<0·0001
	≤14 years	35/764 (4·6%)	70/1471 (4·8%)	0·057*
	15–16 years	274/764 (35·9%)	393/1471 (26·7%)	0·057*
	17–18 years	235/764 (30·8%)	284/1471 (19·3%)	0·057*
Number of lifetime sex partners				
	Reported no vaginal sex	220/817 (26·9%)	724/1469 (49·3%)	<0·0001
	One	207/817 (25·3%)	269/1469 (18·3%)	0·81*
	Two	189/817 (23·1%)	225/1469 (15·3%)	0·81*
	Three or more	201/817 (24·6%)	251/1469 (17·1%)	0·81*
Condom used at last sexual encounter*		333/548 (60·8%)	379/705 (53·8%)	0·013

Data are n/N (%) and exclude missing or unrecorded values, unless otherwise specified.

* Among those who reported ever having vaginal sex.

The prevalence of HPV-16 and HPV-18 was significantly lower in the post-vaccine group (49 [3·2%] of 1538 participants) than the pre-vaccine group (177 [21·6%] of 819), with a relative reduction of 83% (adjusted prevalence ratio 0·17, 95% CI 0·12–0·24; p<0·0001; table 2). When examined individually, there was an 83% relative reduction in HPV-16 (0·17, 0·11–0·25) and an 86% relative reduction in HPV-18 (0·14, 0·08–0·24) in the post-vaccine group. The prevalence of HPV-31 and HPV-45 was also significantly lower in the post-vaccine group (88 [5·7%] of 1538 participants) than the pre-vaccine group (136 [16·6%] of 819), with a relative reduction of 59% (adjusted prevalence ratio 0·41, 95% CI 0·31–0·54; p<0·0001). No reductions in prevalence were noted for other HPV groups. Results remained consistent when the analysis was limited to participants who reported ever having vaginal sex (appendix 1 pp 2–3). We observed modestly higher prevalence ratios for any non-vaccine oncogenic HPV types (adjusted prevalence ratio 1·14, 1·02–1·27) and low-risk HPV-6 and HPV-11 types (1·29, 1·06–1·58) that are not included in the bivalent vaccine, driven mainly by HPV-51 and HPV-6 (table 2; appendix 1 p 4).

**Table 2:** Association between HPV prevalence by groups of HPV types and survey period

	**Pre-vaccine group (n=819)**	**Post-vaccine group (n=1538)**	**Crude PR (95% CI)**	Adjusted PR (95% CI)*	**p value**
HPV-16 and HPV-18	177 (21·6%, 18·9–24·6)	49 (3·2%, 2·4–4·2)	0·15 (0·11–0·20)	0·17 (0·12–0·24)	<0·0001
HPV-16	112 (13·7%, 11·5–16·2)	33 (2·1%, 1·5–3·0)	0·16 (0·11–0·23)	0·17 (0·11–0·25)	<0·0001
HPV-18	89 (10·9%, 8·9–13·2)	18 (1·2%, 0·7–1·9)	0·11 (0·07–0·18)	0·14 (0·08–0·24)	<0·0001
HPV-31 and HPV-45	136 (16·6%, 14·2–19·3)	88 (5·7%, 4·7–7·0)	0·34 (0·27–0·44)	0·41 (0·31–0·54)	<0·0001
HPV-33, HPV-52, and HPV-58	185 (22·6%, 19·9–25·6)	329 (21·4%, 19·4–23·5)	0·95 (0·81–1·11)	1·07 (0·90–1·27)	0·44
HPV-35, HPV-39, HPV-51, HPV-56, HPV-59, and HPV-68	335 (40·9%, 37·6–44·3)	630 (41·0%, 38·5–43·4)	1·00 (0·90–1·11)	1·14 (1·02–1·27)	0·021
HPV-33, HPV-35, HPV-39, HPV-51, HPV-52, HPV-56, HPV-58, HPV-59, and HPV-68	399 (48·7%, 45·3–52·1)	749 (48·7%, 46·2–51·2)	1·00 (0·92–1·09)	1·08 (0·99–1·19)	0·074
Any oncogenic HPV	463 (56·5%, 53·1–59·9)	775 (50·4%, 57·9–52·9)	0·89 (0·83–0·96)	0·96 (0·88–1·03)	0·26
HPV-6 and HPV-11	136 (16·6%, 14·2–19·3)	295 (19·2%, 17·3–21·2)	1·16 (0·96–1·39)	1·29 (1·06–1·58)	0·013

Data are n (%, 95% CI) unless specified otherwise. HPV=human papillomavirus. PR=prevalence ratio.

* Variables included in the adjusted models were age, HIV status, province, currently in school, head of household has an income, smoking status, frequency of alcohol use, uses contraception, history of any sex, and age at first vaginal sex with those that reported no vaginal sex as the reference group. All variance inflation factors were less than 2·5, indicating low multicollinearity.

Among girls living with HIV, those in the post-vaccine group were less likely to report current cigarette smoking, current contraceptive use, or any sexual activity, were diagnosed with HIV at a younger age, had longer ART duration, and were more likely to have an HIV RNA viral load test result ≤50 log_10_ copies per mL in the past median 6 months (IQR 3–12) than those in the pre-vaccine group (table 3). Among participants tested, viral suppression rates were similar.

**Table 3:** Cohort characteristics of adolescent girls living with HIV by survey period

		**Pre-vaccine group (n=248)**	**Post-vaccine group (n=295)**	**p value**
Age		..	..	0·49
	17 years	112/248 (45·2%)	142/295 (48·1%)	..
	18 years	136/248 (54·8%)	153/295 (51·9%)	..
Province of residence		..	..	1·00
	Free State	157/248 (63·3%)	188/295 (63·7%)	..
	Gauteng	30/248 (12·1%)	35/295 (11·9%)	..
	Mpumalanga	30/248 (12·1%)	37/295 (12·5%)	..
	North West	31/248 (12·5%)	35/295 (11·9%)	..
In school		188/247 (76·1%)	239/295 (81·0%)	0·16
Head of household has income		..	..	<0·0001
	No	94/241 (39·0%)	63/295 (21·4%)	..
	Yes	92/241 (38·2%)	190/295 (64·4%)	..
	Unknown	55/241 (22·8%)	42/295 (14·2%)	..
In a relationship		112/248 (45·2%)	122/295 (41·4%)	0·37
Current smoker		32/241 (13·3%)	18/293 (6·1%)	0·0049
Frequency of alcohol use		..	..	0·56
	Never	140/247 (56·7%)	167/275 (60·7%)	..
	Once per month or less	81/247 (32·8%)	85/275 (30·9%)	..
	Two times per month or more	26/247 (10·5%)	23/275 (8·4%)	..
Uses contraception		179/248 (72·2%)	176/295 (59·7%)	0·0022
Ever had any sex		168/247 (68·0%)	155/282 (55·0%)	0·0021
Age at first vaginal sex		..	..	<0·0001
	Reported no vaginal sex	82/243 (33·7%)	157/285 (55·1%)	0·73*
	≤14 years	19/243 (7·8%)	19/285 (6·7%)	0·73*
	15–16 years	84/243 (34·6%)	66/285 (23·2%)	0·73*
	17–18 years	58/243 (23·9%)	43/285 (15·1%)	0·73*
Number of lifetime sex partners		..	..	<0·0001
	Reported no vaginal sex	82/247 (33·2%)	157/284 (55·3%)	0·57*
	One	50/247 (20·2%)	34/284 (12·0%)	0·57*
	Two	51/247 (20·6%)	36/284 (12·7%)	0·57*
	Three or more	64/247 (25·9%)	57/284 (20·1%)	0·57*
Condom used at last sexual encounter*		95/150 (63·3%)	75/121 (62·0%)	0·82
Age at HIV diagnosis		16 (8–17)	14 (7–16)	0·0053
	<9 years	58/248 (23·4%)	93/295 (31·5%)	0·0019
	9–14 years	42/248 (16·9%)	59/295 (20·0%)	0·0019
	15–18 years	128/248 (51·6%)	137/295 (46·4%)	0·0019
	Not available	20/248 (8·1%)	6/295 (2·0%)	0·0019
On ART		..	..	<0·0001
	No	16/248 (6·5%)	1/295 (0·3%)	..
	Yes	232/248 (93·5%)	294/295 (99·7%)	..
Time on ART		1 (0–7)	3 (0–10)	<0·0001
	≥2 years	111/248 (44·8%)	174/295 (59·0%)	<0·0001†
	<2 years	119/248 (48·0%)	118/295 (40·0%)	..
	Not on ART	16/248 (6·5%)	1/295 (0·3%)	..
	Not available	2/248 (0·8%)	2/295 (0·7%)	..
Last CD4 count, cells per mm^3^		475 (322–682)	467 (307–738)	0·85
	≤200	18/248 (7·3%)	27/295 (9·2%)	0·64†
	>200	156/248 (62·9%)	176/295 (59·7%)	0·64†
	Not available	74/248 (29·8%)	92/295 (31·2%)	0·64†
Last viral load, log_10_ copies per mL		50·5 (0–759·5)	49 (19–265)	0·79
	≤50	68/248 (27·4%)	114/295 (38·6%)	<0·0001
	>50	68/248 (27·4%)	98/295 (33·2%)	0·49†
	Not available	112/248 (45·2%)	83/295 (28·1%)	0·49†

Data are n/N (%) or median (IQR) and exclude missing or unrecorded values, unless otherwise specified. Difference in median (IQR) between groups is based on the Kruskal–Wallis test, all other differences are based on χ^2^ tests. ART=antiretroviral therapy.

* Among those who reported ever having vaginal sex.

† Among those with available results.

The prevalence of HPV-16 and HPV-18 was significantly lower in participants living with HIV in the post-vaccine group (13 [4·4%] of 295) than those in the pre-vaccine group (73 [29·4%] 248), with a relative reduction of 82% (adjusted prevalence ratio 0·18, 95% CI 0·10–0·32; p<0·0001; table 4). There was an 83% relative reduction in HPV-16 (0·17, 0·08–0·37) and an 85% relative reduction in HPV-18 (0·15, 0·06–0·34) in the post-vaccine group. The prevalence of HPV-31 and HPV-45 was also significantly lower in the post-vaccine group (25 [8·5%] of 295 participants) than the pre-vaccine group (53 [21·4%] of 248), with a relative reduction of 41% (adjusted prevalence ratio 0·49, 95% CI 0·32–0·76; p=0·0015). No reductions in prevalence were noted for other HPV groups (table 4). Results were similar for girls not living with HIV (table 4, figure; appendix 1 p 5). Results of the complete-case analysis were also consistent with the main findings (appendix 1 p 6).

**Table 4:** Association between HPV prevalence by groups of HPV genotypes, survey period, and HIV status

	**Pre-vaccine group**	**Post-vaccine group**	**Crude PR (95% CI)**	Adjusted PR (95% CI)*	**p value**
**Living with HIV**					
HPV-16 and HPV-18	73/248 (29·4%, 24·1–35·4)	13/295 (4·4%, 2·6–7·5)	0·15 (0·9–0·26)	0·18 (0·10–0·32)	<0·0001
HPV-16	42/248 (16·9%, 12·8–22·1)	7/295 (2·4%, 1·1–4·9)	0·14 (0·06–0·31)	0·17 (0·08–0·37)	<0·0001
HPV-18	44/248 (17·7%, 13·5–23·0)	6/295 (2·0%, 0·9–4·5)	0·11 (0·05–0·26)	0·15 (0·06–0·34)	<0·0001
HPV-31 and HPV-45	53/248 (21·4%, 16·7–26·9)	25/295 (8·5%, 5·8–12·3)	0·40 (0·25–0·62)	0·49 (0·32–0·76)	0·0015
HPV-33, HPV-52, and HPV-58	79/248 (31·9%, 26·3–37·9)	83/295 (28·1%, 23·3–33·6)	0·88 (0·68–1·14)	1·07 (0·84–1·35)	0·61
HPV-35, HPV-39, HPV-51, HPV-56, HPV-59, and HPV-68	120/248 (48·4%, 42·2–54·6)	139/295 (47·1%, 41·5–52·8)	0·97 (0·82–1·16)	1·13 (0·97–1·33)	0·12
HPV-33, HPV-35, HPV-39, HPV-51, HPV-52, HPV-56, HPV-58, HPV-59, and HPV-68	144/248 (58·1%, 51·8–64·1)	162/295 (54·9%, 49·2–60·5)	0·95 (0·82–1·10)	1·08 (0·96–1·22)	0·22
Any oncogenic HPV	162/248 (65·3%, 59·2–71·0)	166/295 (56·3%, 50·5–61·8)	0·86 (0·75–0·99)	0·96 (0·87–1·07)	0·47
HPV 6- and HPV-11	61/248 (24·6%, 19·6–30·4)	70/295 (23·7%, 19·2–28·9)	0·96 (0·72–1·30)	1·12 (0·83–1·50)	0·46
**Not living with HIV**					
HPV-16 and HPV-18	96/540 (17·8%, 14·8–21·2)	36/1218 (3·0%, 2·1–4·1)	0·17 (0·11–0·24)	0·18 (0·12–0·27)	<0·0001
HPV-16	64/540 (11·9%, 9·4–14·9)	26/1218 (2·1%, 1·5–3·1)	0·18 (0·12–0·28)	0·20 (0·12–0·33)	<0·0001
HPV-18	42/540 (7·8%, 5·8–10·4)	12/1218 (1·0%, 0·6–1·7)	0·13 (0·07–0·24)	0·14 (0·07–0·28)	<0·0001
HPV-31 and HPV-45	78/540 (14·4%, 11·7–17·7)	61/1218 (5·0%, 3·9–6·4)	0·35 (0·25–0·48)	0·39 (0·27–0·56)	<0·0001
HPV-33, HPV-52, and HPV-58	96/540 (17·8%, 14·8–21·2)	244/1218 (20·0%, 17·9–22·4)	1·13 (0·91–1·39)	1·24 (0·99–1·57)	0·066
HPV-35, HPV-39, HPV-51, HPV-56, HPV-59, and HPV-68	200/540 (37·0%, 33·1–41·2)	484/1218 (39·7%, 37·0–42·5)	1·07 (0·94–1·22)	1·22 (1·06–1·40)	0·0046
HPV-33, HPV-35, HPV-39, HPV-51, HPV-52, HPV-56, HPV-58, HPV-59, and HPV-68	237/540 (43·9%, 39·8–48·1)	579/1218 (47·5%, 44·7–50·4)	1·08 (0·97–1·21)	1·19 (1·05–1·33)	0·0044
Any oncogenic HPV	279/540 (51·7%, 47·5–55·9)	601/1218 (49·3%, 46·5–52·2)	0·96 (0·86–1·05)	1·03 (0·93–1·15)	0·56
HPV-6 and HPV-11	72/540 (13·3%, 10·7–16·5)	223/1218 (18·3%, 16·2–20·6)	1·37 (1·07–1·76)	1·40 (1·07–1·83)	0·014

Data are n/N (%, 95% CI), unless specified otherwise. HPV=human papillomavirus. PR=prevalence ratio. VIF=variance inflation factor.

* Among girls living with HIV, variables included in the adjusted models were head of household has an income, smoking status, current contraceptive use, ever had any sex, and time on antiretroviral treatment. All VIFs were less than 2·1, indicating low multicollinearity. Age at HIV diagnosis was excluded from this model due to multicollinearity (VIF of 4·8). Among those not living with HIV, variables included in the adjusted models were age, province, currently in school, head of household has an income, smoking status, alcohol use, current contraceptive use, ever had any sex, and age at first vaginal sex with those that reported no vaginal sex as the reference group. All VIFs were less than 2·2, indicating low multicollinearity.

**Figure:**
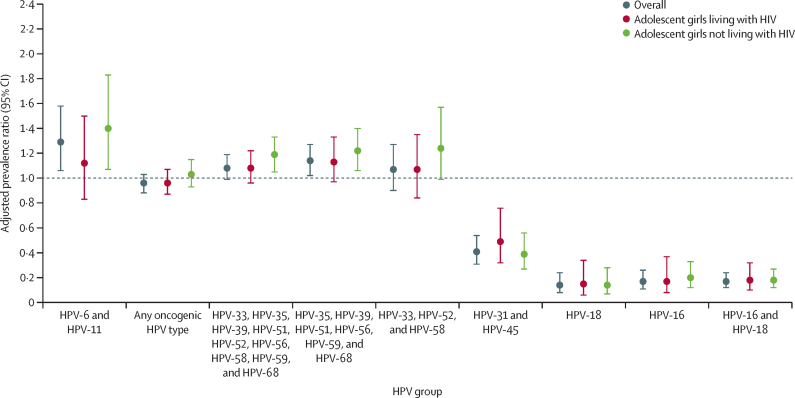
Comparison of HPV prevalence between the pre-vaccine group and post-vaccine group, by HIV status Error bars represent 95% CIs. HPV=human papillomavirus.

When crude HPV prevalence was examined by age at HIV diagnosis, time on ART, and recent viral load, HPV-16 and HPV-18 prevalence was consistently lower in the post-vaccine group than the pre-vaccine group across all subgroups, whereas the prevalence of non-vaccine types remained similar in the pre-vaccine and post-vaccine groups (appendix 1 p 7).

Among the 1538 participants in the post-vaccine group, 324 (21·1%) had identifiers that matched records in the district vaccination registers: 303 (19·7%) had a record of having received two HPV vaccine doses and 21 (1·4%) had a record of receiving only one HPV dose. Among 309 matched participants with a date of vaccination recorded, 176 (57·0%) had received the HPV vaccine before age 12 years, and 305 (98·7%) had received it before age 15 years. Four (1·3%) participants were vaccinated at age 15 years. The median interval between vaccine receipt and survey enrolment was 6 years (IQR 4–8). A further 997 (63·5%) participants were classified as vaccinated based solely on self-report, and the remaining 237 (15·4%) were classified as unvaccinated (appendix 1 p 8).

In the post-vaccine group, girls with register-verified vaccination had the lowest HPV-16 and HPV-18 prevalence (five of 324 [1·5%, 95% CI 0·6–3·7]), followed by those who self-reported vaccination (31 of 977 [3·2%, 2·2–4·5]). The highest prevalence was observed in the unvaccinated group who had no register match and self-reported being unvaccinated or unsure (13 of 237 [5·5%, 3·2–9·2]; appendix 1 p 8).

In the post-vaccine group, HPV-16 and HPV-18 prevalence was 92% lower among register-verified vaccinated girls (adjusted prevalence ratio 0·08, 95% CI 0·03–0·20), 83% lower in the self-reported vaccinated group (0·17, 0·12–0·25), and 74% lower among unvaccinated girls (0·26, 0·15–0·45) than those in the pre-vaccine group. For HPV-31 and HPV-45, prevalence was 80% lower among register-verified vaccinated girls (0·20, 0·10–0·39), 56% lower in the self-reported group (0·44, 0·33–0·60), and 44% lower among unvaccinated girls (0·56, 0·35–0·90). Modest increases or no differences were observed for other HPV groups. Reclassification of all girls without register-confirmed vaccination as unvaccinated, regardless of self-reported status, did not change the results (appendix 1 pp 9–10).

## Discussion

To our knowledge, this is the first documented evidence of the population impact of a two-dose programme, and the first among cohorts who are eligible for routine HPV vaccination in a setting with endemic HIV. Using a repeat cross-sectional study design and recruiting participants from primary health-care clinics, we recorded an 83% relative reduction in the prevalence of vaccine-preventable HPV types 16 and 18 in adolescent girls and young women aged 17–18 years, who were among the first cohorts offered the HPV vaccine in South Africa's HPV school-based vaccination programme. The population impact was the same for girls living with and without HIV, as indicated by similar estimates of relative reductions in analyses stratified by HIV status. Moreover, benefits extended to reductions in the prevalence of vaccine-targeted types among unvaccinated girls (indirect effects) and reductions in circulating HPV types 31 or 45 (cross-protection). Effects were specific to these HPV types, with no reductions for other HPV types, strongly suggesting a genuine programme impact rather than changes in high-risk sexual behaviour.

The magnitude of impact we observed was consistent with that previously reported from three-dose programmes in high-income countries.^7^ A pooled analysis of 23 before-and-after studies of three-dose programmes in 13 countries showed that after 5–8 years of vaccination, the prevalence of HPV-16 or HPV-18 decreased by 83% (adjusted prevalence ratio 0·17, 95% CI 0·11–0·25) among girls aged 13–19 years. A key factor in the strong result achieved by the two-dose programme in South Africa was the very high coverage in the first years of the programme, estimated at over 80%.^13^

Our study also showed that cross-protection against non-vaccine types under a two-dose schedule was similar to that observed in three-dose programmes, with a 54% relative reduction in types 31, 33, and 45 reported in combined analyses (adjusted prevalence ratio 0·46, 95% CI 0·33–0·66).^7^ More recent data have shown that cross-protection is primarily driven by HPV-31 and HPV-45,^21^ which justifies our exclusion of HPV-33 from this cross-protection group in our analyses. Similar to our findings, combined analyses of three-dose programmes also reported modest increases in non-vaccine HPV types, possibly due to changes in sexual activity patterns or unmasking as vaccination reduces vaccine-type HPV infections, allowing previously undetectable coexisting non-vaccine HPV types to be identified during testing.^23^

Another encouraging finding is the level of protection against HPV infection for adolescent girls living with HIV, a population at the highest future risk of cervical cancer. In the 2023 post-vaccine sample, two-thirds of the girls with HIV were diagnosed with HIV between ages 15 years and 18 years, and one-third before age 9 years; the latter probably reflecting perinatal acquisition. Most girls were on ART and had high CD4^+^ cell counts. Provided ART and long-term vaccine effectiveness can be maintained, our study suggests that the benefit of vaccination in cervical cancer prevention is equivalent in those with and without HIV infection. The findings also highlight the importance of early ART initiation and long-term surveillance in vaccinated populations living with HIV to monitor potential waning immunity and the need for boosters. HIV-related immune dysregulation—particularly CD4^+^ T-cell depletion—can impair vaccine response and accelerate antibody decline.^24^ Although early ART mitigates some effects, reduced HPV vaccine effectiveness might still occur over time.^25^ Effectiveness might also depend on dose number and antibody levels achieved before HIV acquisition, which are important considerations as many African countries, including South Africa, adopt single-dose HPV vaccination schedules.^26^ Although few participants in this survey had received a single dose, surveillance data among a one-dose catch-up cohort provide some reassurance, showing no clear differences by HIV status at 2 years post-vaccination.^16^ However, more long-term data are needed.

This study has limitations. The repeated cross-sectional design relies on recruiting similar populations over time, so shifts in underlying population characteristics could bias impact estimates, although it enables pragmatic evaluation without requiring long-term follow-up. Although clinic attendees might not be representative of all adolescents, this approach prioritises repeatability to detect changes between birth cohorts. If vaccine coverage is similar across groups, impacts are likely generalisable, although differences in coverage, timing of vaccine introduction, or population risk profiles could influence observed effects. Incomplete vaccine documentation required reliance on self-report, which might have led to misclassification and underestimation or overestimation of indirect effects. Nonetheless, the prevalence of vaccine-targeted HPV types was consistently lower in 2023 in the post-vaccine group across all vaccination-status definitions. Furthermore, missing values were treated as a separate category, which might have introduced bias. However, analyses excluding missing data showed no meaningful change in the results. Some models included few HPV events, increasing the risk of overfitting; however, effect estimates remained directionally consistent with available evidence, supporting confidence in our findings. The population living with HIV were recruited from clinics, with most on ART. Although South Africa is progressing towards the 95–95–95 targets, adolescents remain less likely to engage in care,^27^ meaning the impact might be lower in populations with lower ART coverage. Finally, although the sample of girls living with HIV was sufficient for estimating overall vaccine effects, it was relatively small, reducing our ability to explore whether HIV-related factors—such as ART timing, immune status, or age at HIV diagnosis (used as a proxy for mode of acquisition)—modify vaccine impact. These factors are interrelated, making independent effects difficult to isolate. Nevertheless, exploratory analyses suggest that vaccine impact might be similar across these subgroups.

In summary, we found a substantial decline in vaccine-type HPV prevalence among South African adolescent girls, with similar reductions in those with and without HIV. Declines in vaccine-type HPV prevalence among unvaccinated girls suggest indirect protection, highlighting the success of the national school-based HPV vaccination programme. These reductions, targeting HPV types causing approximately 70% of cervical cancers, are expected to reduce cervical cancer burden over time. These findings are highly relevant to other sub-Saharan African countries introducing HPV vaccination, particularly in settings with high HIV and cervical cancer burden. As South Africa transitions to a single-dose schedule, continued surveillance—especially among women with HIV—will be crucial to monitor long-term effectiveness and any emerging disparities.

### The HOPE study group

### Contributors

### Equitable partnership declaration

### Data sharing

Data collected for this study might be made available on request. On completion of the study, data will be shared through a general data sharing repository, figshare.com, in accordance with the University of the Witwatersrand's data sharing policies.

## Declaration of interests

DAM has received honoraria, speaking fees, and consultation fees from MSD in the preceding 36 months; none of these were related to this study. All other authors declare no competing interests.

## Supplementary Materials

Supplementary appendix 1

Equitable Partnership Declaration
