# Supplementary material for: Population impact of South Africa's human papillomavirus (HPV) vaccination programme on HPV prevalence in adolescent girls with and without HIV: a repeat cross-sectional study
Source: Lancet Glob Health. 2026 Mar 16;14(4):e570–8. doi: 10.1016/S2214-109X(25)00525-X (PMC13000116; doi:10.1016/S2214-109X(25)00525-X)
Supplement: Supplementary appendix 1 [file mmc1.pdf]

THE LANCET
Global Health

Supplementary appendix 1

This appendix formed part of the original submission and has been peer reviewed. We post it as supplied by the authors.

Supplement to: Machalek DA, Nyemba DC, Travill D, et al. Population impact of South Africa's human papillomavirus (HPV) vaccination programme on HPV prevalence in adolescent girls with and without HIV: a repeat cross-sectional study. *Lancet Glob Health* 2026; **14:** e570–78.

Supplementary appendix

Table of contents

Supplementary Table 1: Cohort characteristics by survey period among participants that reported ever having vaginal sex 2

Supplementary Table 2: Association between HPV prevalence, by groups of HPV types and survey period among participants that reported ever having vaginal sex 3

Supplementary Table 3: Association between HPV prevalence, by individual HPV types and survey period 4

Supplementary Table 4: Cohort characteristics of adolescent girls without HIV by survey period 5

Supplementary Table 5 Adjusted prevalence ratios (95% CIs) from the complete-case sensitivity analysis, presented for the overall population and stratified by HIV status 6

Supplementary Figure 1: Comparison of crude HPV prevalence between pre-vaccine and post-vaccine samples among participants living with HIV, by available clinical characteristics 7

Supplementary Table 6: Crude HPV prevalence by groups of HPV types, survey period and vaccination status* 8

Supplementary Table 7: Cohort characteristics by survey period and vaccination status in the post-vaccine sample 9

Supplementary Table 8: Association between HPV prevalence, by groups of HPV types and vaccination status in the post-vaccine cohort compared with the pre-vaccine sample .. 10

Supplementary Table 1: Cohort characteristics by survey period among participants that reported ever having vaginal sex

		Survey 1	Survey 2	
**Variable**		**Pre-vaccine sample**	**Post-vaccine sample**	
		**N=597**	**N=751**	
		**n (Col %)**	**n (Col %)**	**p-value**
**Age**	17 years	249/597 (41·7)	367/751 (48·9)	0·0009
	18 years	348/597 (58·3)	384/751 (51·1)	
**HIV status**	HIV negative	411/597 (68·8)	618/751(82·3)	<0·0001
	HIV positive	165/597 (27·6)	128/751 (17·0)	
	Unknown	21/597 (3·5)	5/751 (0·7)	
**Province of residence**	Free State	365/597 (61·1)	513/751 (68·3)	0·015
	Gauteng	83/597 (13·9)	92/751 (12·3)	
	Mpumalanga	70/597 (11·7)	82/751 (10·9)	
	North West	79 (13·2)	64/751 (8·5)	
**Currently in school**	No	111/595 (18·7)	89/751 (11·9)	<0·0001
	Yes	484/595 (81·3)	662/751 (88·2)	<0·0001
**Head of household has an income**	No	234/579 (40·4)	130/751 (17·3)	
	Yes	250/579 (43·2)	518/751 (69·0)	
	Don't know	95/579 (16·4)	103/751 (13·7	
**In a relationship**	Yes	334/597 (56·0)	447/751 (59·5)	0·19
**Smoking status**	Current smoker	77/582 (13·2)	47/743 (6·3)	<0·0001
**Frequency of alcohol use**	Once per month or less	290/596 (48·7)	263/706 (37·3)	<0·0001
	Two times per month or more	229/596 (38·4)	374/706 (53·0)	
	Once per month or less	77/596 (12·9)	69/706 (9·8)	
**Current contraceptive use**	Yes	532/597 (89·1)	584/751 (77·7)	<0·0001
**Age at first vaginal sex**	≤ 14 years	35/544 (6·4)	70/747 (9·4)	0·057
	15-16 years	274/544 (50·4)	393/747 (52·6)	
	17-18 years	235/544 (43·2)	284/747 (38·0)	
**Number of lifetime sex partners**	One	207/597 (34·7)	269/745 (36·1)	0·81
	Two	189/597 (31·7)	225/745 (30·2)	
	Three or more	201/597 (33·7)	251/745 (33·7)	
**Condom use at last sexual encounter**	Yes	333/548 (60·8)	379/705 (53·8)	0·013

Data are n/N (%) unless otherwise specified. Unless otherwise specified, percentages exclude missing or unrecorded values.

Supplementary Table 2: Association between HPV prevalence, by groups of HPV types and survey period among participants that reported ever having vaginal sex

HPV type	Crude prevalence<br>Survey 1: Pre-vaccine sample<br>N=597<br>N; % (95% CI)	Crude prevalence<br>Survey 2: Post-vaccine sample<br>N=751<br>N; % (95% CI)	Crude PR (95% CI)	Adjusted PR (95% CI)*	p-value
**HPV 16/18**	159; 26·6 (23·2–30·3)	29; 3·9 (2·7–5·5)	0·14 (0·10–0·21)	0·16 (0·11–0·24)	<0·0001
**HPV 16**	103; 17·3 (14·4–20·5)	19; 2·5 (1·6–3·9)	0·15 (0·09–0·24)	0·15 (0·09–0·25)	<0·0001
**HPV 18**	80; 13·4 (10·9–16·4)	10; 1·3 (0·7–2·5)	0·10 (0·05–0·19)	0·13 (0·06–0·25)	<0·0001
**HPV 31/45**	126; 21·1 (18·0–24·6)	56; 7·5 (5·8–9·6)	0·35 (0·26–0·47)	0·37 (0·27–0·52)	<0·0001
**HPV33/52/58**	160; 26·8 (23·4–30·5)	186; 24·8 (21·8–28·0)	0·92 (0·77–1·11)	1·04 (0·86–1·27)	0·68
**HPV35/39/51/56/59/68**	294; 49·3 (45·3–53·3)	375; 49·9 (46·4–53·5)	1·01 (0·91–1·13)	1·12 (1·00–1·26)	0·068
**HPV33/35/39/51/52/56/58/59/68**	348; 58·3 (54·3–62·2)	439; 58·5 (54·9–61·9)	1·00 (0·92–1·10)	1·09 (0·99–1·20)	0·070
**Any oncogenic HPV**	405; 67·8 (64·0–71·5)	456; 60·7 (57·2–64·2)	0·90 (0·83–0·97)	0·95 (0·88–1·04)	0·28
**HPV 6/11**	122; 20·4 (17·4–23·9)	169; 22·5 (19·7–25·6)	1·10 (0·90–1·36)	1·15 (0·91–1·44)	0·24

[#] Variables included in the adjusted models were: age, HIV status, province, currently in school, head of household has an income, smoking status, frequency of alcohol use, age at first vaginal sex and condom use at last sex. All VIFs were below 1·5, indicating low multicollinearity.
PR: Prevalence ratios
CI: Confidence Intervals

Supplementary Table 3: Association between HPV prevalence, by individual HPV types and survey period

HPV type	Survey 1: Pre-vaccine sample	Survey 2: Post-vaccine sample			
	N=819	N=1,538			
	N; % (95% CI)	N; % (95% CI)	Crude PR (95% CI)	Adjusted PR (95% CI)*	p-value
HPV 31	68 (8·3; 6·6–10·4)	32 (2·1; 1·5–2·9)	0·25 (0·17–0·38)	0·32 (0·20–0·50)	<0·0001
HPV 45	75 (9·2; 7·4–11·3)	58 (3·8; 2·9–4·9)	0·41 (0·30–0·57)	0·48 (0·33–0·69)	<0·0001
HPV 33	34 (4·2; 3·0–5·8)	36 (2·3; 1·7–3·2)	0·56 (0·36–0·89)	0·66 (0·39–1·12)	0·12
HPV 35	98 (12·0; 9·9–14·4)	123 (8·0; 6·7–9·5)	0·67 (0·52–0·86)	0·87 (0·65–1·15)	0·33
HPV 39	100 (12·2; 10·1–14·6)	163 (10·6; 9·2–12·2)	0·87 (0·69–1·10)	1·03 (0·80–1·34)	0·80
HPV 51	77 (9·4; 7·6–11·6)	204 (13·3; 11·7–15·1)	1·41 (1·10–1·81)	1·53 (1·17–2·00)	0·0021
HPV 52	111 (13·6; 11·4–16·1)	182 (11·8; 10·3–13·6)	0·87 (0·70–1·09)	1·03 (0·81–1·31)	0·79
HPV 56	92 (11·2; 9·2–13·6)	172 (11·2; 9·7–12·9)	1·00 (0·78–1·26)	1·21 (0·93–1·57)	0·16
HPV 58	89 (10·9; 8·9–13·2)	154 (10·0; 8·6–11·6)	0·92 (0·72–1·18)	1·11 (0·85–1·46)	0·45
HPV 59	77 (9·4; 7·6–11·6)	154 (10·0; 8·6–11·6)	1·07 (0·82–1·38)	1·23 (0·93–1·64)	0·15
HPV 68	80 (9·8; 7·9–12·0)	120 (7·8; 6·6–9·3)	0·80 (0·61–1·05)	0·95 (0·71–1·28)	0·74
HPV 6	96 (11·7; 9·7–14·1)	210 (13·7; 12·0–15·5)	1·16 (0·93–1·46)	1·31 (1·02–1·69)	0·031
HPV 11	57 (7·0; 5·4–8·9)	112 (7·3; 6·1–8·7)	1·05 (0·77–1·42)	1·24 (0·89–1·74)	0·21

[#] Variables included in the adjusted models were: age, HIV status, province, currently in school, head of household has an income, smoking status, frequency of alcohol use, current contraceptive use, history of any sex, and age at first vaginal sex with those that reported no vaginal sex as the reference group. All VIFs were below 2·4, indicating low multicollinearity.
PR: Prevalence ratios
CI: Confidence Intervals

Supplementary Table 4: Cohort characteristics of adolescent girls without HIV by survey period

		Survey 1	Survey 2	
**Variable**		**Pre-vaccine sample**	**Post-vaccine sample**	
		**N=540**	**N=1218**	
		**n (Col %)**	**n (Col %)**	**p-value**
**Age**	17 years	269/540 (49·8)	703/1218 (57·7)	0·0021
	18 years	271/540 (50·2)	515/1218 (42·3)	
**Province of residence**	Free State	337/540 (62·4)	868/1218 (71·3)	<0·0001
	Gauteng	80/540 (14·8)	119/1218 (9·8)	
	Mpumalanga	44/540 (8·2)	111/1218 (9·1)	
	North West	79/540 (14·6)	120/1218 (9·9)	
**Currently in school¶**	Yes	470/538 (87·4)	1,137/1218 (93·4)	<0·0001
**Head of household has an income¶**	Yes	202/526 (38·4)	219/1218 (18·0)	<0·0001
	No	231/526 (43·9)	806/1218 (66·2)	
	Don’t know	93/526 (17·7)	193/1218 (15·9)	
**In a relationship**	Yes	252/540 (46·7)	598/1218 (49·1)	0·35
**Smoking status¶**	Current smoker	51/525 (9·7)	57/1208 (4·7)	<0·0001
**Frequency of alcohol use¶**	Never	297/540 (55·0)	511/1129 (45·3)	<0·0001
	Once per month or less	185/540 (34·3)	520/1129 (46·1)	
	Two times per month or more	58/540 (10·7)	98/1129 (8·7)	
**Current contraceptive use**	Yes	439/540 (81·3)	737/1218 (60·5)	<0·0001
**Ever had any sex**	Yes	417/538 (77·5)	742/1163 (63·8)	<0·0001
**Age at first vaginal sex$**	Reported no vaginal sex	128/492 (26·0)	550/1164 (47·3)	<0·0001
	≤ 14 years	14/492 (2·9)	51/1164 (4·4)	0·0049$
	15-16 years	180/492 (36·6)	324/1164 (27·8)	
	17-18 years	170/492 (34·6)	239/1164 (20·5)	
**Number of lifetime sex partners$**	Reported no vaginal sex	128/539 (23·8)	550/1163 (47·3)	<0·0001
	One	149/539 (27·6)	233/1163 (20·0)	0·82$
	Two	132/539 (24·5)	187/1163 (16·1)	
	Three or more	130/539 (24·1)	1931163 (16·6)	
**Condom use at last sexual encounter$**	Yes	225/377 (59·7)	300/579 (51·8)	0·017

Data are n/N (%) unless otherwise specified. Unless otherwise specified, percentages exclude missing or unrecorded values. $ Among those who reported ever having vaginal sex.

Supplementary Table 5 Adjusted prevalence ratios (95% CIs) from the complete-case sensitivity analysis, presented for the overall population and stratified by HIV status

HPV type	Overall	Living with HIV	Not living with HIV
	**Adjusted PR (95% CI)**	**Adjusted PR (95% CI)**	**Adjusted PR (95% CI)**
**HPV 16/18**	0·18 (0·13–0·26)	0·17 (0·10–0·30)	0·17 (0·11–0·28)
**HPV 16**	0·18 (0·12–0·29)	0·16 (0·07–0·36)	0·21 (0·12–0·36)
**HPV 18**	0·14 (0·08–0·25)	0·14 (0·06–0·33)	0·12 (0·06–0·27)
**HPV 31/45**	0·43 (0·32–0·58)	0·51 (0·32–0·80)	0·43 (0·29–0·62)
**HPV33/52/58**	1·13 (0·95–1·36)	1·12 (0·86–1·45)	1·31 (1·02–1·67)
**HPV35/39/51/56/59/68**	1·19 (1·07–1·35)	1·17 (0·99–1·38)	1·27 (1·10–1·47)
**HPV33/35/39/51/52/56/58/59/68**	1·11 (1·00–1·25)	1·08 (0·96–1·22)	1·23 (1·08–1·39)
**Any oncogenic HPV**	1·00 (0·93–1·08)	0·96 (0·87–1·07)	1·06 (0·95–1·19)
**HPV 6/11**	1·34 (1·09–1·66)	1·12 (0·83–1·50)	1·43 (1·07–1·91)

aPR (95% CI): adjusted prevalence ratio (95% confidence intervals)
Among overall population variables included in the adjusted models were: age, HIV status, province, currently in school, head of household has an income, smoking status, frequency of alcohol use, current contraceptive use, history of any sex, and age at first vaginal sex with those that reported no vaginal sex as the reference group.
Among girls living with HIV variables included in the adjusted models were: head of household has an income, smoking status, current contraceptive use, ever had any sex and time on antiretroviral treatment.
Among those not living with HIV variables included in the adjusted models were: age, province, currently in school, head of household has an income, smoking status, alcohol use, current contraceptive use, ever had any sex, and age at first vaginal sex with those that reported no vaginal sex as the reference group.

Supplementary Figure 1: Comparison of crude HPV prevalence between pre-vaccine and post-vaccine samples among participants living with HIV, by available clinical characteristics

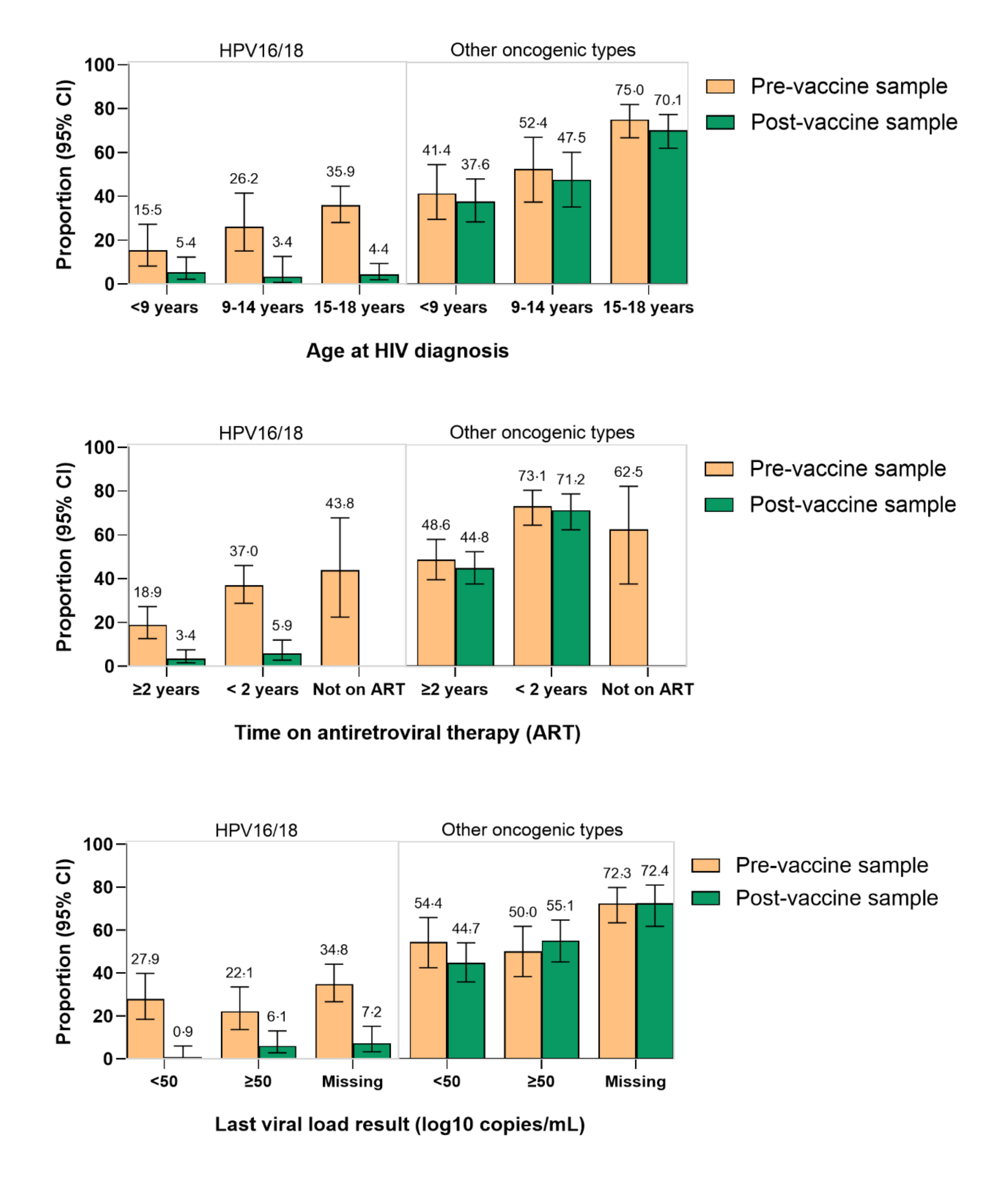

CI: Confidence Intervals. See Table 3 for the total number of participants in each stratum. There was only one person not on ART in the post-vaccine sample who was negative for HPV16/18 and other oncogenic HPV types

Supplementary Table 6: Crude HPV prevalence by groups of HPV types, survey period and vaccination status*

HPV type	Survey 1<br>Pre-vaccine sample<br>N=819<br>n; % (95% CI)	Survey 2: Vaccinated[1]<br>District registers<br>n=324<br>n; % (95% CI)	Survey 2: Vaccinated[2]<br>Self-report alone<br>n=977<br>n; % (95% CI)	Survey 2: Unvaccinated[3]<br>Main analysis<br>n=237<br>n; % (95% CI)	Survey 2: Unvaccinated[4]<br>Sensitivity<br>n=1,214<br>n; % (95% CI)
**HPV16/18**	177; 21·6 (18·9–24·6)	5; 1·5 (0·6–3·7)	31; 3·2 (2·2–4·5)	13; 5·5 (3·2–9·2)	44; 3·6 (2·7–4·8)
**HPV16**	112; 13·7 (11·5–16·2)	5; 1·5 (0·6–3·7)	17; 1·7 (1·1–2·8)	11; 4·6 (2·6–8·2)	28; 2·3 (1·6–3·3)
**HPV18**	89; 10·9 (8·9–13·2)	0; 0 (0–1·1)	15; 1·5 (0·9–2·5)	3; 1·3 (0·4–3·9)	18; 1·5 (0·9–2·3)
**HPV31/45**	136; 16·6 (14·2–19·3)	9; 2·8 (1·5–5·3)	60; 6·1 (4·8–7·8)	19; 8·0 (5·2–12·2)	79; 6·5 (5·3–8·0)
**HPV33/52/58**	185; 22·6 (19·9–25·6)	79; 24·4 (20·0–29·4)	195; 20·0 (17·6–22·6)	55; 23·2 (18·3–29·0)	250; 20·6 (18·4–23·0)
**HPV35/39/51/56/59/68**	335; 40·9 (37·6–44·3)	133; 41·1 (35·8–46·5)	383; 39·2 (36·2–42·3)	114; 48·1 (41·8–54·5)	497; 40·9 (38·2–43·7)
**HPV33/35/39/51/52/56/58/59/68**	399; 48·7 (45·3–52·1)	161; 49·7 (44·3–55·1)	456; 46·7 (43·6–49·8)	132; 55·7 (49·3–61·9)	588; 48·4 (45·6–51·3)
**Any oncogenic HPV**	463; 56·5 (53·1–59·9)	161; 49·7 (44·3–55·1)	478; 48·9 (45·8–52·1)	136; 57·4 (51·0–63·5)	614; 50·6 (47·8–53·4)
**HPV6/11**	136; 16·6 (14·2–19·3)	51; 15·7 (12·2–20·0)	193; 19·8 (17·4–22·4)	51; 21·5 (16·4–27·2)	244; 20·1 (17·9–22·5)

CI: Confidence Intervals.
Participants in the post-vaccine sample were classified into four vaccination status groups: (1) vaccinated with at least one register-confirmed dose; (2) vaccinated by self-report only (no matching register); (3) unvaccinated (main analysis): no register match and self-reported unvaccinated or unsure; and (4) unvaccinated (sensitivity analysis): no register match, regardless of self-report.

Supplementary Table 7: Cohort characteristics by survey period and vaccination status in the post-vaccine sample

		Survey 1	Survey 2: Vaccinated[1]	Survey 2: Vaccinated[2]	Survey 2: Unvaccinated[3]	Survey 2: Unvaccinated[4]		
		Pre-vaccine sample	District registers	Self-report alone	Main analysis	Sensitivity		
**Variable**		**N=819**	**n=324**	**n=977**	**n=237**	**n=1214**		
		**n (%)**	**n (%)**	**n (%)**	**n (%)**	**n (%)**	**p-value***	**p-value****
**Age**	17 years	396/819 (48·4)	202/324 (62·4)	553/977 (56·6)	102/237 (43·0)	655/1214 (54·0)	<0·0001	<0·0001
	18 years	423/819 (51·6)	122/324 (37·7)	424/977 (43·4)	135/237 (57·0)	559/1214 (46·1)		
**HIV status**	HIV negative	540/819 (65·9)	256/324 (79·0)	778/977 (79·6)	184/237 (77·6)	962/1214 (79·2)	<0·0001	<0·0001
	HIV positive	248/819 (30·3)	64/324 (19·8)	182/977 (18·6)	49/237 (20·7)	231/1214 (19·0)		
	Unknown	31/819 (3·8)	4/324 (1·2)	17/977 (1·7)	4237 (1·7)	21/1214 (1·7)		
**Province of residence**	Free State	506/819 (61·8)	261/324 (80·6)	695/977 (71·1)	124/237 (52·3)	819/1214 (67·5)	<0·0001	<0·0001
	Other	313/819 (38·2)	63/324 (19·4)	282/977 (28·9)	113/237 (47·7)	395/1214 (32·5)		
**Currently in school¶**	Yes	684/816 (83·8)	306/324 (94·9)	887/977 (90·8)	205/237 (86·5)	1092/1214 (90·0)	<0·0001	<0·0001
**Head of household has an income¶**	No	310/798 (38·8)	52/324 (16·1)	192/977 (19·7)	42/237 (17·7)	234/1214 (19·3)	<0·0001	<0·0001
	Yes	336/798 (42·1)	224/324 (69·1)	627/977 (64·2)	159/237 (67·1)	786/1214 (64·7)		
	Don't know	152/798 (19·0)	48/324 (14·8)	158/977 (16·2)	36/237 (15·2)	194/1214 (16·0)		
**In a relationship**	Yes	372/819 (45·4)	147/324 (45·4)	452/977 (46·3)	127/237 (53·6)	579/1214 (47·7)	0·15	0·54
**Smoking status¶**	Current smoker	86/797 (10·8)	19/320 (5·9)	43/970 (4·4)	13/236 (5·5)	56/1206 (4·6)	<0·0001	<0·0001
**Frequency of alcohol use¶**	Never	455/818 (55·6%)	142/296 (48·0)	458/912 (50·2)	94/220 (42·7)	552/1132 (48·8)	<0·0001	0·00001
	Once per month or less	275/818 (33·6%)	129/296 (43·6)	383/912 (42·0)	101/220 (45·9)	484/1132 (42·8)		
	Two times per month or more	88/818 (10·8%)	25/296 (8·5)	71/912 (7·8)	25/220 (11·4)	96/1132 (8·5)		
**Current contraceptive use**	Yes	640/819 (78·1%)	195/324 (60·2)	590/977 (60·4)	134/237 (56·5)	724/1214 (59·6)	<0·0001	<0·0001
**Ever had any sex**	Yes	607/816 (74·4%)	180/307 (58·6)	564/934 (60·4)	160/227 (70·5)	724/1161 (62·4)	<0·0001	<0·0001
**Age at first vaginal sex$**	Reported no vaginal sex	220/764 (28·8%)	165/305 (54·1)	470/943 (49·8)	89/223 (39·9)	559/1166 (48·0)	<0·0001	<0·0001
	≤ 14 years	35/764 (4·6%)	13/305 (4·3)	44/943 (4·7)	13/223 (5·8)	57/1166 (4·9)	0·16$	0·81$
	15-16 years	274/764 (35·9%)	81/305 (26·6)	249/943 (26·4)	63/223 (28·3)	312/1166 (26·8)		
	17-18 years	235/764 (30·8%)	46/305 (15·1)	180/943 (19·1)	58/223 (26·0)	238/1166 (20·4)		
**Number of lifetime sex partners$**	Reported no vaginal sex	220/817 (26·9%)	165/304 (54·3)	470/942 (49·9)	89/223 (39·9)	559/1165 (48·0)	<0·0001	<0·0001
	One	207/817 (25·3%)	45/304 (14·8)	181/942 (19·2)	43/223 (19·3)	224/1165 (19·2)	0·28$	0·81$
	Two	189/817 (23·1%)	43/304 (14·1)	147/942 (15·6)	35/223 (15·7)	182/1165 (15·6)		
	Three or more	201/817 (24·6%)	51/304 (16·8)	144/942 (15·3)	56/223 (25·1)	200/1165 (17·2)		
**Condom use at last sexual encouter$**	Yes	333/548 (60·8%)	75/134 (56·0)	250/444 (56·3)	54/127 (42·5)	304/571 (53·2)	0·027	0·039

Participants in the post-vaccine sample were classified into four vaccination status groups: (1) vaccinated with at least one register-confirmed dose; (2) vaccinated by self-report only (no matching register); (3) unvaccinated (main analysis): no register match and self-reported unvaccinated or unsure; and (4) unvaccinated (sensitivity analysis): no register match, regardless of self-report.

Data are n/N (%) unless otherwise specified. Unless otherwise specified, percentages exclude missing or unrecorded values. $ Among those who reported ever having vaginal sex. *p-value compares the pre-vaccine proportion to post-vaccine proportion by vaccination groups 1, 2, and 3; **p-value compares the pre-vaccine proportion to the post-vaccine proportion by vaccination groups 1 and 4.

Supplementary Table 8: Association between HPV prevalence, by groups of HPV types and vaccination status in the post-vaccine cohort compared with the pre-vaccine sample

**Survey 2: Vaccinated[1] (district registers)**			
**HPV type**	**Crude PR (95% CI)**	**Adjusted PR (95% CI)***	**p-value**
**HPV 16/18**	0·07 (0·03–0·17)	0·08 (0·03–0·20)	<0·0001
**HPV 16**	0·11 (0·05–0·27)	0·11 (0·05–0·28)	<0·0001
**HPV 18**	N/A$	N/A$	
**HPV 31/45**	0·17 (0·09–0·32)	0·20 (0·10–0·39)	<0·0001
**HPV33/52/58**	1·08 (0·86–1·36)	1·32 (1·04–1·68)	0·021
**HPV35/39/51/56/59/68**	1·00 (0·86–1·17)	1·10 (0·94–1·29)	0·23
**HPV33/35/39/51/52/56/58/59/68**	1·02 (0·90–1·16)	1·14 (1·00–1·30)	0·044
**Any oncogenic HPV**	0·88 (0·78–1·00)	0·96 (0·85–1·09)	0·56
**HPV 6/11**	0·95 (0·71–1·27)	1·11 (0·82–1·51)	0·51
**Survey 2: Vaccinated[2] (self-report only)**			
**HPV type**	**Crude PR (95% CI)**	**Adjusted PR (95% CI)***	**p-value**
**HPV 16/18**	0·15 (0·10–0·21)	0·17 (0·12–0·25)	<0·0001
**HPV 16**	0·13 (0·08–0·21)	0·14 (0·08–0·23)	<0·0001
**HPV 18**	0·14 (0·08–0·24)	0·18 (0·11–0·32)	<0·0001
**HPV 31/45**	0·37 (0·28–0·49)	0·44 (0·33–0·60)	<0·0001
**HPV33/52/58**	0·88 (0·74–1·06)	1·05 (0·87–1·26)	0·63
**HPV35/39/51/56/59/68**	0·96 (0·86–1·07)	1·05 (0·94–1·18)	0·38
**HPV33/35/39/51/52/56/58/59/68**	0·96 (0·87–1·06)	1·07 (0·97–1·18)	0·16
**Any oncogenic HPV**	0·87 (0·79–0·94)	0·95 (0·87–1·03)	0·20
**HPV 6/11**	1·19 (0·98–1·45)	1·38 (1·12–1·70)	0·028
**Survey 2: Unvaccinated[3] (main analysis)**			
**HPV type**	**Crude PR (95% CI)**	**Adjusted PR (95% CI)***	**p-value**
**HPV 16/18**	0·25 (0·15–0·44)	0·26 (0·15–0·45)	<0·001
**HPV 16**	0·34 (0·19–0·62)	0·30 (0·16–0·56)	<0·001
**HPV 18**	0·12 (0·04–0·36)	0·14 (0·04–0·45)	0·001
**HPV 31/45**	0·48 (0·31–0·76)	0·56 (0·35–0·90)	0·017
**HPV33/52/58**	1·03 (0·79–1·34)	1·19 (0·91–1·56)	0·20
**HPV35/39/51/56/59/68**	1·18 (1·01–1·37)	1·21 (1·04–1·41)	0·014
**HPV33/35/39/51/52/56/58/59/68**	1·14 (1·00–1·31)	1·26 (1·11–1·43)	<0·001
**Any oncogenic HPV**	1·02 (0·90–1·15)	1·10 (0·98–1·24)	0·10
**HPV 6/11**	1·30 (0·97–1·73)	1·40 (1·04–1·88)	0·026
**Survey 2: Unvaccinated[4] (sensitivity)**			
**HPV type**	**Crude PR (95% CI)**	**Adjusted PR (95% CI)***	**p-value**
**HPV 16/18**	0·17 (0·12–0·23)	0·19 (0·14–0·27)	<0·001
**HPV 16**	0·17 (0·11–0·25)	0·17 (0·11–0·27)	<0·001
**HPV 18**	0·14 (0·08–0·22)	0·17 (0·10–0·29)	<0·001
**HPV 31/45**	0·39 (0·30–0·51)	0·47 (0·35–0·62)	<0·001
**HPV33/52/58**	0·91 (0·77–1·08)	1·08 (0·90–1·28)	0·42
**HPV35/39/51/56/59/68**	1·00 (0·90–1·11)	1·08 (1·01–1·11)	0·011
**HPV33/35/39/51/52/56/58/59/68**	0·99 (0·91–1·09)	1·11 (1·01–1·21)	0·030
**Any oncogenic HPV**	0·89 (0·82–0·97)	0·97 (0·90–1·05)	0·52
**HPV 6/11**	1·21 (1·00–1·46)	1·38 (1·13–1·69)	0·0028

CI: Confidence Intervals.
$Calculation could not be performed due to the absence of HPV18-positive results.
Participants in the post-vaccine sample were classified into four vaccination status groups: (1) vaccinated with at least one register-confirmed dose; (2) vaccinated by self-report only (no matching register); (3) unvaccinated (main analysis): no register match and self-reported unvaccinated or unsure; and (4) unvaccinated (sensitivity analysis): no register match, regardless of self-report. *Variables included in the adjusted models were: age, HIV status, province, currently in school, head of household has an income, smoking status, frequency of alcohol use, current contraceptive use and history of any sex All VIFs were below 1.5, indicating low multicollinearity.
