# Supplementary material for: Population impact of South Africa's human papillomavirus (HPV) vaccination programme on HPV prevalence in adolescent girls with and without HIV: a repeat cross-sectional study
Source: Lancet Glob Health. 2026 Mar 16;14(4):e570–8. doi: 10.1016/S2214-109X(25)00525-X (PMC13000116; doi:10.1016/S2214-109X(25)00525-X)
Supplement: Equitable Partnership Declaration [file mmc2.pdf]

THE LANCET
Global Health

Supplementary appendix 2

This Equitable Partnership Declaration (EPD) was submitted by the authors, and we reproduce it as supplied. It has not been peer reviewed. *The Lancet*'s editorial processes have not been applied to the EPD.

Supplement to: Machalek DA, Nyemba DC, Travill D, et al. Population impact of South Africa's human papillomavirus (HPV) vaccination programme on HPV prevalence in adolescent girls with and without HIV: a repeat cross-sectional study. *Lancet Glob Health* 2026; **14:** e570–78.

Equitable Partnership Declaration

If any questions do not apply to your study, please indicate "N/A" for "not applicable.
For more information on how to complete this form see the Information for Authors document.

Researcher considerations

1. Please detail the involvement that researchers who are based in the country or countries of study had during a) study design; b) clinical study processes, such as processing blood samples, prescribing medication, or patient recruitment; c) data interpretation; and d) manuscript preparation, commenting on all aspects. If they were not involved in any of these aspects, please explain why.

   This should include a thorough description of their leadership roles in the study. Are local researchers named in the author list or the acknowledgements, or are they not mentioned at all (and, if not, why)? Please also describe the involvement of early career researchers based in the location of the study. Some of this information might be repeated from the Contributors section in the manuscript. Note: we adhere to [ICMJE authorship criteria] for naming authors on a paper.

**a) Study design:** Researchers based in South Africa played a central role in the design and development of the study. Their extensive experience in clinical trials of new HIV prevention technologies, observational cohort studies of HPV infection, and implementation research on the HPV vaccine programme informed the scientific and operational framework of the study. They contributed to identifying research priorities, selecting appropriate study populations and sites, developing study protocols and data collection tools, and ensuring that study procedures were contextually relevant and aligned with local healthcare systems. Their in-depth understanding of the local epidemiology, healthcare infrastructure, and community dynamics ensured that the study design was both scientifically rigorous and culturally appropriate.
**b) Clinical study processes**: The study team worked closely with the Department of Basic Education and Department of Health, implementing a catch-up campaign of the single dose of the bivalent vaccine which was offered to approximately 4800 Grade 10 girls across 66 public high schools in Lejweleputswa district in 2019 based on Department of Health procedures. The South African investigators supervised the study team conducting the surveys and trained clinical staff in participant assessment and sample collection. Study staff were embedded within Department of Health sites and contributed to seeing clinic participants for their presenting complaint in accordance with national guidelines. The study investigators based in South Africa were responsible for interpretation of HPV DNA test results and follow up and referral of participants with positive results.
**c) Data interpretation:** The South African investigators designed the survey data collection instruments with input from all investigators. The South African investigators trained the study team in survey administration and designed the database for capture of survey information. The South African investigators had oversight of data cleaning and quality management. The South African investigators also worked with the Department of Health to capture vaccination status information from Department of Health registers held at facilities. The South African team were responsible for matching participants with their vaccination register status and verifying vaccination status. All data management activities, including data cleaning, coding, validation, and preparation for analysis were performed locally by the study team, with support from international investigators. Local researchers also participated in the statistical analysis,

interpretation of findings, and contextualization of results within the national HPV vaccination programme.
**d) Manuscript preparation:** Members from the team were involved in developing the analysis plan, supported data analysis and reviewed and revised the manuscript.

2. How was funding used to remunerate and enhance the skills of researchers in the countries of study? And how was funding used to improve research infrastructure at the study sites?

   Potentially effective investments into long-term skills and opportunities within local institutions could include training or mentorship in analytical techniques and manuscript writing, opportunities to lead all or specific aspects of the study, financial remuneration rather than requiring volunteers, and other professional development and educational opportunities.

   Improvements to research infrastructure could include funding extended trial designs (eg, platform trials), establishment of long-term contracts for research staff, building research facilities, and setting up local control of funding allocation.

**Skills:** Funding was strategically utilized to strengthen both human capacity and research infrastructure at the study sites. A significant portion supported capacity-building activities within participating clinics, including training healthcare providers with educational material and improvement in data collection and study procedures to enhance the quality and consistency of implementation. Some funds were allocated to support early career investigators through mentorship, conference attendance, and manuscript development workshops. This support enabled emerging researchers to build scientific writing skills, contribute to manuscripts, and participate in national and international conferences.
**Research infrastructure:** Investments were also made to improve research infrastructure, including procurement of laboratory equipment including freezers and portable refrigerators and data collection equipment.

3. How did you safeguard the researchers who implemented the study?

   Please describe how you guaranteed safe working conditions for study staff, including provision of appropriate personal protective equipment, protection from violence, and prevention of overworking.

All study staff received training on Standard Operating Procedures and the Manual of Procedures, including detailed work instructions for each study activity. Appropriate personal protective equipment such as gloves, masks, laboratory coats, and hand sanitizers, was provided to all staff. Clinical and laboratory personnel received additional training on biosafety, specimen handling, and waste management to reduce the risk of exposure to infectious agents. The study team also worked with community advisory boards (CABs) at each site to promote community safety and acceptance. The CABs provided valuable input on local context, cultural sensitivities, and safety considerations, fostering trust and buy-in from participating communities and helping to identify and mitigate potential risks early in implementation. To prevent overworking, staff schedules were designed to balance workload and ensure adequate rest periods. Regular monitoring of workload and wellbeing was conducted to minimize burnout, with flexible scheduling and access to psychosocial support

where needed. The study was implemented during the COVID19 pandemic and all activities then were governed by institutional procedures to maximise staff and participant safety. Health care workers were prioritised for vaccine access when vaccines became available.

Benefits to the communities and regions of study

4. How does the study address the research and policy priorities of its location?

How were the local priorities determined and then used to inform the research question? Who decided which priorities to take forward? Which elements of the study address those priorities?

The study team worked closely with the Department of Basic Education and Department of Health. Members of the study team had worked on the assessment of the implementation of the HPV vaccine programme in South Africa which informed the design of the evaluation of both two and single dose of HPV vaccine. The study was presented to stakeholders at the national department of health who endorsed the importance of the study and provided letters of support for each of the provincial departments of health involved. The Department of Health staff are co-authors on this manuscript

5. How will research products be shared in the community of study?

For instance, will you be providing written or oral layperson summaries for non-academic information sharing? Will study data be made available to institutions in the region(s) of study? The Lancet Global Health *encourages authors to translate the summary (abstract) into relevant languages after paper editing; do you intend to translate your summary?*

Data generated from the project was presented regularly to community advisory boards and professional bodies (NITAG, RITAG and WHO IVR, PATH and SAGE) as well as the South African Department of Health.

6. How were individuals, communities, and environments protected from harm?

a) *How did you ensure that sensitive patient data were handled safely and respectfully? Was there any potential for stigma or discrimination against participants arising from any of the procedures or outcomes of the study?*

Confidentiality of all study participants was maintained by identifying data through a unique identification number in a secure database. Only named investigators and study staff had access to the data. All participants of eligible age were offered an opportunity to join the study and there was therefore no potential stigma or discrimination for either participating or not participating in the study. If participants declined participation they were still offered clinical services consistent with national health guidelines by the study nurse.

b) *Might any of the tests be experienced as invasive or culturally insensitive?*

Girls were asked to self-collect a vaginal swab for HPV testing. Step-by-step illustrated instructions were provided to ensure proper sample collection and participant comfort. The research team had extensive experience conducting STI surveys involving self-sampling among adolescents and women in South Africa. The self-collection procedure was non-invasive, well-tolerated, and culturally acceptable within the study communities.

c) *How did you determine that work was sensitive to traditions, restrictions, and considerations of all cultural and religious groups in the study population?*

The study team actively engaged with Community Advisory Boards at each study site, receiving valuable guidance on the local context and cultural norms helping the team to adapt study procedures in ways that were respectful and acceptable to participants. This collaboration fostered trust and buy-in from the communities and allowed potential cultural or religious concerns to be identified and addressed early in the implementation process.

d) *Were biowaste and radioactive waste disposed of in accordance with local laws?*

No radioactive waste was produced during the study. Standard operating procedures for waste management were followed at all study sites. Staff were trained on proper segregation, labelling, storage, and disposal of hazardous materials, and compliance was regularly monitored to ensure the safety of staff, participants, and the surrounding community

e) *Were any structures built that would have impacted members of the community or the environment (such as handwashing facilities in a public space)? If so, how did you ensure that you had appropriate community buy-in?*

Not applicable

f) *How might the study have impacted existing health-care resources (such as staff workloads, use of equipment that is typically employed elsewhere, or reallocation of public funds)?*

Sites selected were affiliated with the South Africa STI surveillance network and they offer routine HIV testing and counselling, as well as a range of other sexual and reproductive health services. They have a strong history of research collaboration with the research team and experience incorporating self-collection of vaginal swabs among young women. The study activities were integrated smoothly into existing clinic workflows without significant additional burden on staff or infrastructure. No reallocation of public funds or diversion of essential healthcare resources was required. Where necessary, additional study staff and supplies were provided to ensure that routine healthcare services were not affected.

7. Confirm that local ethics review was sought, and please provide the approval number. If not sought, please explain why.

The project was approved by the University of the Witwatersrand Human Research Ethics Committee (HREC #181005), and the University of New South Wales HREC (#181-005).

<u>Secondary analyses</u>

8. Have the data analysed in your study been extracted from another source, such as a national survey, rather than being directly collected by the authors of this paper?

~~Yes~~ / No / ~~N/A~~

If the authors of this paper were not involved in data collection, how were the findings interpreted with sufficient contextual knowledge?
The Lancet Global Health *believe contextual understanding is crucial for informed data analysis and interpretation*.

Authors of this paper were involved in data collection

---

9. Finally, please provide the title (eg, Dr/Prof, Mr/Mrs/Ms/Mx), name, and email address of an author who can be contacted about this statement.

Name: Prof Sinead Delany-Moretlwe
Email: sdelany@wrhi.ac.za
